# Crosstalk between circulating peroxisome proliferator-activated receptor gamma, adipokines and metabolic syndrome in obese subjects

**DOI:** 10.1186/1758-5996-5-79

**Published:** 2013-12-12

**Authors:** Khadijeh Mirzaei, Arash Hossein-nezhad, Seyed Ali Keshavarz, Fariba Koohdani, Ali Akbar Saboor-Yaraghi, Saeed Hosseini, Mohammad Reza Eshraghian, Mahmoud Djalali

**Affiliations:** 1 Cellular and Molecular Nutrition Department, School of Nutritional Science and Dietetics, Tehran University of Medical Sciences, Tehran, Iran; 2 Tehran University of Medical Sciences, Tehran, Iran; 3 Department of Medicine, Section of Endocrinology, Nutrition, and Diabetes, Vitamin D, Skin and Bone Research Laboratory, Boston University Medical Center, Boston, Massachusetts, United States of America; 4 Clinical Nutrition Department, School of Nutritional Science and Dietetics, Tehran University of Medical Sciences, Tehran, Iran; 5 Department of Biostatistics and Epidemiology, School of Public Health, Tehran University of Medical Sciences, Tehran, Iran

**Keywords:** Metabolic syndrome, Circulating PPARγ, Adipokines, Obesity

## Abstract

**Background:**

Peroxisome proliferator-activated receptor gamma (PPARγ) has direct and indirect function in adipokines production process. We aimed to assess the possible influence of circulating PPARγ on relative risk of metabolic syndrome and also examine the association between circulating PPARγ and adipokines levels among obese subjects.

**Methods:**

A total of 96 obese subjects (body mass index (BMI) ≥30) were included in the current cross-sectional study. We assessed the body composition with the use of Body Composition Analyzer *BC-418MA - Tanita*. The MetS (metabolic syndrome) was defined based on the National Cholesterol Education Program Adult Treatment Panel III. All baseline blood samples were obtained following an overnight fasting. Serum concentrations of adipokines including Retinol binding protein 4 (RBP4), omentin-1, vaspin, progranulin, nesfatin-1 and circulating PPARγ was measured with the use of an enzyme-linked immunosorbent assay method. Statistical analyses were performed using software package used for statistical analysis (SPSS).

**Results:**

We found main association between circulating PPARγ and body composition in obese population. The risk of metabolic syndrome in subjects with higher concentration of PPARγ was 1.9 fold in compared with lower concentration of PPARγ after adjustment for age, sex and BMI. There was significant association between PPARγ and adipokines, specially nesfatin-1 and progranulin. Defined adipokines pattern among participants demonstrated the markedly higher concentration of vaspin, RBP4 and nesfatin-1 in participants with MetS compared to non-MetS subjects.

**Conclusions:**

It appears all of studied adipokines might have association with PPARγ level and might simultaneously be involve in some common pathway to make susceptible obese subjects for MetS.

## Background

The possibility of having metabolic irregularities, including Metabolic Syndrome (MetS), develops with the level of obesity. Obese subjects with MetS had significantly increased risk of mortality compared to without MetS [1]. Although, the underlying mechanisms of obesity's influence on MetS development are not completely understood; but compelling evidences suggested the main effect of adipokines in this procedure [2,3].

Adipokines, with important endocrine functions expresses and secretes factors from adipose tissue [4]. Among numerous secreted adipokines, some of them including nesfatin-1 [5], retinol binding protein 4 (RBP4) [6], omentin-1 [7], vaspin [8], and progranulin [9], provide an extensive network of communication both within adipose tissue and are implicated directly in the pathologies associated with metabolic syndrome [10].

The MetS was defined based on the National Cholesterol Education Program Adult Treatment Panel III criteria as presenting at least three of the following components: 1) waist circumferences 102 cm or greater in men or 88 cm or greater in women; 2) triglycerides 1.7 mmol/liter (150 mg/dl) or greater; 3) high density lipoprotein (HDL) cholesterol less than 1.03 mmol/liter (40 mg/dl) in men or less than 1.29 mmol/liter (50 mg/dl) in women; 4) blood pressure 130/85 mm Hg or greater or current use of antihypertensive medications; or 5) fasting plasma glucose 6.1 mmol/liter (110 mg/dl) or greater [11].

The most important determinant of adipokine secretion depends on mature adipocyte size and circulating levels of adipokines verified by average size of adipocytes [12]. Compelling evidences demonstrated the essential role of Peroxisome proliferator-activated receptor gamma (PPARγ) in the formation [13] and size [14] of adipocytes. PPARγ as a member of the nuclear receptor superfamily of ligand-activated transcription factor has direct [15] and indirect [13,14,16] function in adipokines production process. Moreover, there are some evidences that activation of PPARγ pathway [17] through involvement on changes of adipokines could affect on metabolic profiles in animal model of metabolic syndrome studies [18]. In humans, PPARγ is expressed by peripheral cells such as lymphocytes and monocytes [19-22]. The widespread tissue distribution of PPARγ [23] and unknown pathway of the target tissue(s) of circulating synthetic agonists of PPARγ [24] suggests an involvement of the nuclear receptor in multiple peripheral processes. It has been reported that there are balance between nuclear hormone receptor superfamily including PPARγ and proper lipid ligand and target PPARγ drug in peripheral circulation [24,25]. In view of the fact that assessment of circulating PPARγ ligands could clarify some pathway of PPARγ function, so it seems that evaluation of PPARγ concentration similar to its agonist concentration in plasma would be benefit to find its correlation with some important process in metabolism and adipokines secretion. Although, PPARγ genotypes considered as modulator factor in MetS risk in previous studies [26-28], but there was not any reports regarding to association between circulating PPARγ and susceptibility to MetS in obesity. Accordingly, we design current study to assess the possible influence of circulating PPARγ on relative risk of metabolic syndrome and also examine the association between various levels of PPARγ and different pattern of circulating adipokines among obese subjects.

## Methods

### Study population

A total of 96 obese subjects were included in the current cross-sectional study from October of 2011 to January of 2012. The proportion of the men was 18.75% (n = 18) and that of the women was 81.25% (n = 78) in current study's population. All of participants were obese (body mass index (BMI) ≥30). The study was approved by the local ethics committee of the Tehran University of Medical Sciences. Patients were selected according to the defined inclusion criteria which were: BMI ≥30, age 20–50 years, absence of any acute or chronic inflammatory disease, no medical history of hypertension, no alcohol or drug abuse, no use of PPARγ agonist or antagonist, vitamin, dietary, herbal or omega-3 supplements for at least 3 months prior to the study period and not being pregnant or menopause. Exclusion criteria were: history of any condition affecting inflammatory markers such as known cardiovascular diseases, thyroid diseases, malignancies, current smoking, diabetes mellitus, sustained hypertension, heart failure, acute or chronic infections, and hepatic or renal diseases. Sedentary subjects, with restrictive diet and non-normolipidemic were also excluded of the study. First step for analyzing, we categorized all of participants according to median of PPARγ concentration to low and high concentration of PPARγ groups. We next used regression model for calculating the possible risk of MetS according to PPARγ level. All participants gave written informed consent before any study procedure was performed. We performed all of measurements including blood pressure, biochemical and hormonal test, resting metabolic rate and body composition analysis for all of study's participants. Insulin resistance (IR) was calculated by homeostasis model assessment (HOMA). The HOMAIR was calculated according to IRHOMA = [Fasting Plasma Glucose (mmol/L) × Fasting Plasma Insulin (mIU/L)] /22.5 [29]. The BAI is calculated as: (100^x^ hip circumference in m/ height in m ^x^ √height)-18. The waist circumference (WC) was measured at the midpoint between the lower border of the rib cage and the iliac crest by using a flexible inch tape.

### Blood pressure measurement

We measured the blood pressure by Automatic Inflate Blood Pressure Monitor (Samsung BA507S automatic digital blood pressure monitor, Samsung America, Inc.) according to *manufacturer's* instructions in the sitting position of all participants. The blood pressure of all participants was measured after 15-min rest in the chair-seated position by the same person.

### Complete body composition analysis

We assessed the body composition of all cases with the use of Body Composition Analyzer *BC-418MA - Tanita* (United Kingdom). This equipment is designed send out a very weak electric current to measure the impedance (electrical resistance) of the body. Therefore, in principle, subjects were barefoot when they were assessed by this device. Moreover, since impedance fluctuates in accordance with the distribution of the body fluid, we followed all of the following instructions for an accurate measurement. To prevent a possible discrepancy in measured values, we avoided taking measurements after vigorous exercise and waited until the subject was sufficiently rested. To prevent inaccurately low body fat percentage measurements and other measurement errors, we always held both arms straight down when taking measurements. As changes in body-water distribution and body temperature can have a major impact on measurements, they were performed in the morning in a fasting condition (always urinating before taking measurements, etc.) to get a more accurate result of the measurements every single time. The device calculates body fat percentage, fat mass, and fat free mass and predicts muscle mass on the basis of data using Bioelectrical Impedance Analysis (BIA). The main outputs of device are BMI, Fat%, Fat mass, FFM, TBW and visceral fat levels. Through the use of 8 electrodes, the Body Composition Analyzer makes it possible to show separate body composition mass for the right arm, the left arm, the trunk, the right leg and the left leg. We were reported trunk fat along with other important body composition components including fat percent, fat mass, free fat mass and visceral fat in current study.

### RMR measurements

Measurements were performed on all subjects by professional nutritionists using a standard protocol that described in details previously [30]. Resting Metabolic Rate (RMR) was measured by means of the MetaCheck™ (Korr Medical Technologies, Salt Lake City, Utah), an instrument designed to measure RMR using indirect calorimetry. Indirect calorimetry is a method of calculating metabolic rate from the measured the amount of oxygen consumed by the body. Using the MetaCheck mouthpiece, the individual being tested breathes in room air and the gas the person breathes out, is conveyed to the MetaCheck through the breathing hose. The MetaCheck analyzes the volumetric flow and oxygen concentration of the expired gas to determine the amount of oxygen consumed by the body due to metabolism. RMR was measured by indirect calorimetry following an overnight period of 10–12 hour fasting. Subjects were required to fast and remain in a resting state for 12 hours prior to the test and to abstain from smoking ≥ 4 hour before the commencement of the procedure although the ideal interval was 12 h so that to ensure the body was in a resting and post-absorptive state. Patients were instructed to rest in supine position on a mattress for 15 minutes and then they underwent the measurement for a period of 20 minutes. However, the first 5 minutes was not included and only the last 15 minutes were used to calculate RMR.

### Definition of the MetS

The MetS was defined based on the National Cholesterol Education Program Adult Treatment Panel III criteria [11] described completely in previous section [11].

### Biochemical parameters and hormonal assay

Patients fasted for 12 hours before peripheral venous blood was collected from the patients. All baseline blood samples were obtained between 8:00 and 10:00 am. Serum was centrifuged, aliquoted and stored at a temperature of −80ºC. All samples were analyzed by means of a single assay. Glucose Oxidase Phenol 4-Aminoantipyrine Peroxidase (GOD/PAP) method was used for the measurement of fasting serum glucose, and, triglyceride levels were measured by Glycerol-3-phosphate oxidase Phenol 4-Aminoantipyrine Peroxidase (GPO-PAP) method. Total cholesterol levels were measured by Enzymatic Endpoint method, and direct high and low density lipoprotein was measured by enzymatic clearance assay. Fasting serum glucose and lipid profile measurements were done with the use of Randox laboratories kit (Hitachi 902). Liver function test including Aspartate transaminase *(*AST*)*, alkaline phosphatase (ALP) and alanine aminotranferease *(*ALT*)* were measured using an automatic analysis system (Autoanalyzer; Hitachi Ltd, Tokyo, Japan) with Randox laboratories kit. All inter-assay calculated coefficients of variation were within the normal range of enzymatic kits data sheets. Serum Hyper sensitivity C-reactive protein (hsCRP), was measured by means of a imonoturbidimetric assay (High sensitivity assay, by Hitachi 902). Serum insulin concentrations were measured by enzyme-linked immunosorbent assay (ELISA) method (Human insulin ELISA kit, DRG Pharmaceuticals, GmbH, Germany) minimum detectable concentration was 1.76 μlU/ml, Intra CV was 2.19% and Inter CV was 4.4%.

### Circulating adipokines measurements

Serum concentrations of all adipokines were measured in triplicate and 10 replicates per ELISA plate were used as internal quality controls. RBP4 in serum samples was measured by competitive enzyme-linked immunosorbent assay (ELISA) (AdipoGen, Seoul, Korea) and inter- and intra-assay variability were 4.2% and 4.5%, respectively (*Cat. No*. R0822EK). Serum Omentin 1 [Intelectin-1 (human) ELISA Kit] was measured using an ELISA (Enzo Life Sciences; sensitivity: 0.4 ng/ml; reference range: 0.5 –32 ng/ml inter-assay variability: 4.61%; intra-assay variability: 5.2%) (*Cat. No*. APO-54 N-034). Vaspin (human) ELISA Kit (Enzo Life Sciences; sensitivity: 0.01 ng/ml; inter-assay variability: 5.8%; intra-assay variability: 6.5%) *(Cat. No.* ALX-850-375). Serum progranulin was measured with the use of an ELISA method (AdipoGen; Seoul, Korea; sensitivity: 32 pg/ml; inter-assay variability: 4.7%; intra-assay variability: 3.79%) (*Cat. No.* AG-45A-0018EK-KI01). Circulating PPARγ was assayed by Human peroxisome proliferator-activated receptor γ (PPAR-γ) ELISA Kit (CUSABIO BIOTECH, China) sensitivity: 19.53 pg/ml.; detection range: 78.13 pg/ml-5000 pg/ml, inter-assay precision: 6.8%; intra-assay precision: 5.6%) (*Cat. No.* CSB-E08623h). Circulating nesfatin-1 was assayed by Human Nesfatin-1 ELISA Kit (CUSABIO BIOTECH, China) sensitivity: 7.8 pg/ml; detection range: 31.25 pg/ml-2000 pg/ml, inter-assay precision: 7.8%; intra-assay precision: 6.6%) (*Cat. No.* CSB-E15050h).

### Statistical analyses

Normal distribution of data was assured using Kolmogrov-Smirnov. Baseline characteristics and anthropometric measurements of obese participants according to low and high PPARγ level were assessed by Independent- Samples T Test. Categorical variables were compared using chi-square. We used Binary Logistic regression model for calculating the possible risk of MetS according to PPARγ level as only covariates in model 1, and after included age and gender as covariates in model 2; age, gender and BMI as covariates in model 3; and age, gender, BMI and finally body composition components (fat mass and free fat mass) as covariates in model 4. We used Bivariate correlations analysis to find correlation among various adipokines and PPARγ. Finally, we performed the factor analysis (Principal Component Analysis) for defining the pattern of adipokines change in obese subjects and then examine its effect on MetS risk. Data were expressed as mean ± Sd. Error Mean (SEM). The level of significance was set at a probability of ≤0.05 for all tests. Statistical analyses were performed using SPSS version 18.0 (SPSS Inc., Chicago, IL).

## Results

### Baseline characteristics of obese participants according to circulating PPARγ

Baseline characteristics and anthropometric measurements of obese participants according to circulating PPARγ level was demonstrated in Table 1. As shown in this table we found significant differences on weight (pvalue = 0.003), BMI (pvalue = 0.013), fat mass (pvalue = 0.05), Free fat mass (FFM) (pvalue = 0.018), trunk fat (pvalue = 0.008), TG (pvalue = 0.025), RMR (pvalue = 0.008) and WC (pvalue = 0.001) among different categorized levels of circulating PPARγ. Our results demonstrated no significant differences in fat percent, visceral fat, FBS, total cholesterol, HDL and LDL cholesterol, evaluated liver enzymes, hs-CRP, insulin, HOMA-IR, BAI, and blood pressure (P > 0.05) between high and low levels of circulating PPARγ.

**Table 1:** Baseline characteristics and anthropometric measurements of obese participants according to circulating PPARγ level

**Characteristics**	**Low circulating PPARγ (Mean ± SEM) ¶**†	**High circulating PPARγ (Mean ± SEM) ¶**†	**95% confidence interval of the difference (Lower- Upper)**	**P value†**
Age (year)	40.68 ± 1.65	37.44 ± 1.69	−1.47 to 7.95	0.176
Weight(kg)	88.54 ± 1.64	97.92 ± 2.56	−15.41 to-3.35	0.003*
BMI (kg/m2)	34.26 ± 0.56	36.42 ± 0.64	−3.86 to −0.46	0.013*
Fat (%)	40.73 ± 0.86	40.82 ± 0.98	−2.70 to 2.51	0.945
Fat mass (kg)	36.27 ± 1.14	39.72 ± 1.30	−6.88 to 0.003	0.050*
FFM (kg)	52.48 ± 1.13	58.21 ± 2.08	−10.43 to −1.01	0.018*
Visceral Fat (kg)	10.57 ± 0.53	11.48 ± 0.51	−2.38 to 0.55	0.221
Trunk Fat	18.09 ± 0.52	20.37 ± 0.65	−3.95 to −0.60	0.008*
FBS (mg/dl)	103.13 ± 3.73	113.11 ± 6.56	−24.90 to 4.93	0.191
TG (mg/dl)	130.90 ± 8.14	162.65 ± 11.18	−59.26 to −4.22	0.025*
Total Chol (mg/dl)	188.41 ± 4.97	195.91 ± 4.50	−20.84 to 5.85	0.267
HDL Chol (mg/dl)	49.93 ± 1.30	47.46 ± 1.45	−1.40 to 6.34	0.210
LDL Chol (mg/dl)	100.40 ± 3.39	103.93 ± 3.02	−12.57 to 5.50	0.439
AST (IU/L)	16.73 ± 1.03	19.56 ± 1.14	−5.89 to 0.22	0.069
ALT(IU/L)	14.42 ± 1.59	16.45 ± 1.76	−6.75 to 2.68	0.395
ALP(IU/L)	179.50 ± 6.67	185.22 ± 6.55	−24.32 to12.87	0.542
Hs-CRP (mg/l)	3.11 ± 0.41	3.68 ± 0.47	−1.81 to 0.67	0.368
Insulin (μlU/ml)	16.99 ± 1.03	18.36 ± 1.24	−4.57 to 1.85	0.402
HOMA-IR	4.43 ± 0.38	5.31 ± 3.52	−2.21 to 0.44	0.19
RMR (kcal/24 h)	1542.77 ± 56.79	1880.97 ± 84.04	−538.98 to-137.41	0.001*
BAI	39.22 ± 0.75	39.40 ± 0.86	−2.45 to 2.10	0.88
WC (cm)	97.87 ± 1.31	105.26 ± 1.60	−11.51 to −3.26	0.001*
SBP (mmhg)	124.95 ± 3.90	123.06 ± 2.98	−7.85 to11.62	0.702
DBP (mmhg)	86.65 ± 1.89	83.71 ± 2.07	−2.63 to 8.53	0.296

*P-value ≤0.05 are significant.

†Independent- Samples T Test.

¶Mean plus and minus Standard Error Mean.

†n of participants in low circulating PPARγ group = 48, n of participants in high circulating PPARγ group = 48.

BMI, body mass index; FFM, free fat mass; FBS, fasting blood sugar; TG, triglyceride, LDL, low density lipoprotein; HDL, high density lipoprotein; AST, Aspartate transaminase; ALT, alanine aminotransferase; ALP, Alkaline phosphatase; hsCRP, High sensitivity c-reactive protein, HOMA-IR, Homeostasis Model of Assessment - Insulin Resistance; RMR, resting metabolic rate; BAI, Body Adiposity Index; WC, Waist Circumference, SBP, systolic blood pressure, DBP, diastolic blood pressure.

### Circulating PPARγ in the obese subjects and the risk of MetS and its components

Results of logistic regression model demonstrated the main effect of circulating PPARγ on the risk of metabolic syndrome and its components (Table 2). We found the 1.901 increased risk of metabolic syndrome in subjects with higher concentration of PPARγ in compared with lower concentration of PPARγ after adjustment for age, sex and BMI (pvalue = 0.037, 95% CI from 1.041 to 3.473). We also found the 1.88 increased risk of metabolic syndrome in subjects with higher concentration of PPARγ in compared with lower concentration of PPARγ after adjustment for age, sex , BMI and body composition components (pvalue = 0.04, 95% CI from 1.01 to 3.49).

**Table 2:** Risk of Metabolic syndrome and its components may modify by circulating PPARγ in the obese subjects

**Metabolic syndrome and its components**	**Odd ratio**	**95% CI**	**P**
**Metabolic syndrome‡**			
**Model 1‖**	**1.85**	1.07-3.20	**0.02**
**Model 2¶**	**2.05**	1.14-3.67	**0.01**
**Model 3†**	**1.90**	1.04-3.47	**0.03**
**Model 4§**	**1.88**	1.01-3.49	**0.04**
**Abdominal Obesity (WC)**			
**Model 1**	**3.889**	1.49-10.14	**0.005**
**Model 2**	**5.069**	1.66-15.43	**0.004**
**Model 3**	**4.820**	1.30-17.75	**0.018**
**Model 4**	**5.199**	1.07-25.26	**0.04**
Hyperglycemia*			
**Model 1**	1.47	0.77-2.79	0.23
**Model 2**	1.55	0.80-2.99	0.18
**Model 3**	1.42	0.71-2.81	0.31
**Model 4**	1.39	0.67-2.88	0.37
**Hypertension**			
**Model 1**	1.22	0.71-2.10	0.46
**Model 2**	1.49	0.71-3.14	0.28
**Model 3**	1.33	0.62-2.87	0.46
**Model 4**	1.15	0.51-2.61	0.72
**Low HDL**			
**Model 1**	1.70	0.96-3.02	0.06
**Model 2**	1.71	0.95-3.06	0.07
**Model 3**	1.63	0.90-2.95	0.10
**Model 4**	1.62	0.89-2.95	0.11
**Hypertriglyceridemia**			
**Model 1**	1.636	0.933-2.869	0.086
**Model 2**	1.706	0.957-3.042	0.070
**Model 3**	1.593	0.884-2.870	0.121
**Model 4**	1.545	0.84-2.83	0.16

Logistic regression model for circulating PPARγ effect on Risk of Metabolic syndrome and its components.

Total n = 96, n of men =18, n of women = 78, mean of age = 39.06, mean of BMI = 35.34.

The significant value presented by bold format.

‡The MetS was defined based on the National Cholesterol Education Program Adult Treatment Panel III criteria as presenting at least three of the following components: 1) waist circumferences 102 cm or greater in men or 88 cm or greater in women; 2) triglycerides 1.7 mmol/liter (150 mg/dl) or greater; 3) HDL cholesterol less than 1.03 mmol/liter (40 mg/dl) in men or less than 1.29 mmol/liter (50 mg/dl) in women; 4) blood pressure 130/85 mm Hg or greater or current use of antihypertensive medications; or 5) fasting plasma glucose 6.1 mmol/liter (110 mg/dl) or greater.

‖Step 1 in Logistic regression model; MetS entered as dependent and categorized PPARγ (low and high concentration of PPARγ based on median) entered as covariates as a first model.

¶Age, gender entered on Step 1.

†Body Mass Index entered on Step 2.

**§**Body composition components including fat mass and FFM entered on Step 3.

MetS, metabolic syndrome; WC, waist circumferences; HDL, high density lipoprotein.

Analysis of the effect of PPARγ level on the relative risk of MetS components demonstrated the significant effect of circulating PPARγ on abdominal obesity. Accordingly, the relative risk of abdominal obesity was increased 4.82 in subjects with higher concentration of PPARγ in compared with lower concentration of PPARγ after adjustment for age, sex and BMI (pvalue = 0.018, 95% CI from 1.30 to 17.75). Considering to other components of MetS, there is not significant association between hyperglycemia and hypertension relative risk and PPARγ level. We found no significant association between the increased relative risk to low HDL (*p*value = 0.071, 95% CI from 0.956 to 3.069) and hypertriglyceridemia (*p*value = 0.070, 95% CI from 0.957 to 3.042) with high level of PPARγ after adjustment for age and gender.

### Correlation between circulating adipokines and PPARγ, Adipokines levels between low/high concentrations of PPARγ and Adipokines pattern in MetS

We first examined the correlation between circulating adipokines and PPARγ (Table 3). Our results demonstrated positive correlation between circulating PPARγ and nesfatin-1 level (r = 0.275, pvalue = 0.008). We also observed the positive significant correlation between circulating PPARγ and progranulin (r = 0.32, pvalue = 0.032). Consider to correlation of RBP4 (r = −0.353, pvalue = 0.051), omentin-1 (r = 0.289, pvalue = 0.054) and vaspin (r = −0.261, pvalue = 0.084) levels with PPARγ concentration; there was not significant correlation between them. We couldn’t find significant correlation between HOMA-IR as an insulin resistance index and PPARγ and measured adipokines level in our study (pvalue >0.05).

**Table 3:** Correlation between circulating adipokines and PPARγ

**Adipokinesζ**		**Nesfatin-1**	**RBP4**	**Omentin**	**Progranulin**	**Vaspin**	**PPARγ**
Nesfatin-1	Pearson Correlation	1					
	Sig. (2-tailed)	.					
RBP4	Pearson Correlation	−0.201	1				
	Sig. (2-tailed)	0.278	.				
Omentin	Pearson Correlation	0.171	−0.132	1			
	Sig. (2-tailed)	0.263	0.470				
Progranulin	Pearson Correlation	0.022	−0.238	0.379(*)	1		
	Sig. (2-tailed)	0.887	0.190	**0.012**	.		
	Sig. (2-tailed)	0.994	0.408	0.234	0.130		
Vaspin	Pearson Correlation	0.253	0.099	0.363(*)	0.238	1	
	Sig. (2-tailed)	0.094	0.589	**0.014**	0.119	.	
PPARγ	Pearson Correlation	0.275(**)	−0.353	0.289	0.327(*)	0.261	1
	Sig. (2-tailed)	**0.008**	0.051	0.054	**0.032**	0.084	.

**Correlation is significant at the 0.01 level (2-tailed).

*Correlation is significant at the 0.05 level (2-tailed).

ζCorrelation analysis performed by log 10 adipokines levels.

Pearson correlation analysis performed between circulating nesfatin-1, RBP4, omentin, progranulin, vaspin and PPARγ.

Total n = 96, n of men =18, n of women = 78, mean of age = 39.06, mean of BMI = 35.34.

The significant value presented by bold format.

RBP4, retinol binding protein 4; PPARγ, Peroxisome proliferator-activated receptor gamma.

**Figure 1:**
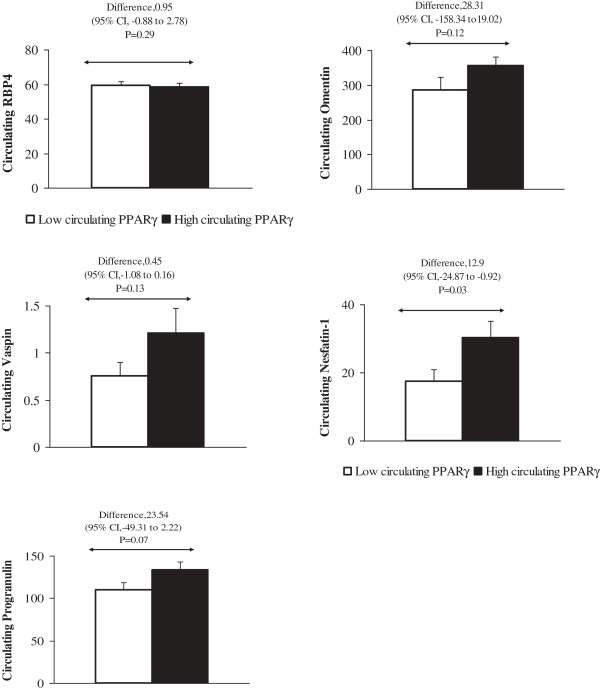
**Independent- Samples T Test analysis performed to detect differences in circulating nesfatin-1, RBP4, omentin, progranulin, vaspin between low/high concentrations of PPARγ.** Total n = 96, n of men =18, n of women = 78, mean of age = 39.06, mean of BMI = 35.34, RBP4, retinol binding protein 4; PPARγ, Peroxisome proliferator-activated receptor gamma.

We next compared the concentration of adipokines between categorized PPARγ levels in low/high groups; our results demonstrated the significant higher concentration of nesfatin-1 in group with high level of PPARγ. There are not significant differences in RBP4 (pvalue = 0.29), omentin-1 (pvalue = 0.12), and vaspin (pvalue = 0.13) levels between high vs, low PPARγ concentration groups. We found not significant differences in circulating progranulin between groups with diverse level of PPARγ (pvalue = 0.07) (Figure 1).

**Figure 2:**
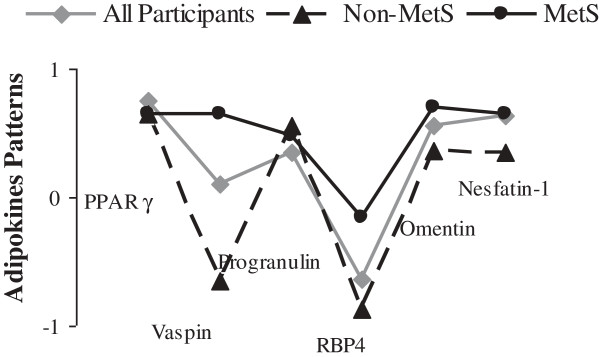
**Factor analysis (Principal Component Analysis) performed for defining the pattern of adipokines change in obese subjects with and without MetS.** The markedly higher concentration of vaspin, RBP4 and nesfatin-1 was seen in participants with MetS compared to non-MetS subjects. Total n = 96, n of men = 18, n of women = 78, mean of age = 39.06, mean of BMI = 35.34, MetS, metabolic syndrome; RBP4, retinol binding protein 4; PPARγ, Peroxisome proliferator-activated receptor gamma.

For completing our analysis consider finding pattern of adipokines changes in MetS; we do the factor analysis. Accordingly, we design principle component analysis to extract one pattern of adipokines levels among participants with MetS and healthy (Figure 2). We found markedly higher concentration of vaspin, RBP4 and nesfatin-1 in participants with MetS compared to non-MetS subjects.

## Discussion

This study demonstrated the main association between circulating PPARγ and body composition in obese population. The risk of metabolic syndrome in subjects with higher concentration of PPARγ was higher in compared with lower concentration of PPARγ after adjustment for age, sex and BMI. We found significant association between PPARγ and adipokines, specially nesfatin-1 and progranulin. Also, according to defined adipokines pattern among participants, we found markedly higher concentration of vaspin, RBP4 and nesfatin-1 in participants with MetS compared to non-MetS subjects.

PPARγ is a ligand-activated transcription factor that considered as a main regulator of adipocyte differentiation [15]. We found the higher levels of weight, BMI, fat mass, FFM and trunk fat among participants with higher concentration of PPARγ. The effect of PPARγ agonist on average fat cell size in the epididymal fat pad reported from animal model study. Results of mentioned study revealed the smaller average fat cell size in untreated *PPARγ+/−* animals compared with wild-type (WT) littermates and was significantly increased after treatment with PPARγ agonist to values comparable to those of controls [31]. Evidences of Kubota et al. [32] study on PPARγ receptor-deficient mice did not display adipocyte hypertrophy. It has been suggested that the primary action of troglitazone as PPARγ agonist may be to increase the number of small adipocytes in white adipose tissues, presumably via PPARγ [14]. It has been shown that moderate reduction of PPARγ with a retinoid X receptor (RXR) antagonist or a PPARγ antagonist decreases triglyceride (TG) content in white adipose tissue, skeletal muscle and liver. These inhibitors changed the some adipokines effects, which increases fatty acid combustion and energy dissipation, thereby ameliorating HF diet-induced obesity [33]. The simultaneous markedly difference in TG level and fat composition between groups with various circulating PPARγ in our research may be related to explained mechanism by Kadowaki et al. [33] study.

It has been demonstrated that the agonist of PPARγ, rosiglitazone, improves the metabolic profile and changes plasma levels of adipokines [18]. So, it appears may be the increased PPARγ level had beneficial effect on metabolic profiles in animal model of MetS. Current study's findings demonstrated that the obese subjects with higher concentration of PPARγ were more susceptible to categorize in MetS group. We observed that in obese people who had more concentration of PPARγ the risk of MetS was increasing in compare to obese people who had lower concentration of PPARγ. In compare to animal model of MetS, the observed raised concentration of PPARγ may act as compensatory mechanism to affect on adipokines levels and other metabolism profile to improve the some MetS outcomes.

There are evidences [34] that a 50% reduction in content of PPARγ receptor did not result in insulin resistance, as one might predict, but rather led to an increase in insulin sensitivity. As a result, it has been suggested that PPARγ deficiency might prevent or attenuate the insulin resistance associated with obesity and other factors. Therefore, due to relative PPARγ deficiency mitigates some physiological causes of insulin resistance, so make a condition as therapeutic maneuver with aimed to produce the same effect as PPARγ deficiency. Thus, this status could be of clinical value in the treatment of insulin resistance [31] as strongly contributor of obesity and other components of the MetS [35].

Our results demonstrated no significant differences in HOMA-IR values between groups with low and high level of PPARγ. However, HOMA-IR has been widely used in various studies, but hardly has consensus on the cut-off points for classification of insulin resistance [36]. An existing hesitation is the clinical value of HOMA-IR for application in clinical prediction of metabolic disorders [36]. The major limitation of the use of HOMA-IR index in studies is that the model applies values calculated from lean young adults of Caucasian origin as standard to other subjects [37,38]. Utilization of values for other population including older adults [39] or obese subjects would probably be different from those documented for previous studies. So, it seems that obese individuals are known to be reasonably more insulin resistant [40]. The ethnicity also considered as a main factor in the etiology of insulin resistance [41]. Previous published results considering cut-off points for HOMA-IR in healthy subjects of our studied community demonstrated values less than observed in current study [36] that likelihood was related to increased insulin resistance in obese individuals. Therefore, the not significant differences in insulin resistance between groups may explain through increased of HOMA-IR value in all of participants and not have defined cut-off points for studied groups.

It has been demonstrated that PPARγ play a key role in the regulation of inflammation and other immune responses [42]. Although, some studies have suggested the contrary findings considering PPARγ role in inflammation status, but it is reported that PPAR activation is associated with anti-inflammatory responses [43]. Despite these evidences, our results demonstrated no significant differences in hs-CRP and liver enzymes between different categorized level of PPARγ groups.

Nesfatin-1 has been implicated in appetite regulation, weight loss and/or malnutrition [44]. Endogenous pancreatic islet nucleobindin 2 (NUCB2)/nesfatin is altered in diabetes and diet-induced obesity [45]. Previous studies observed positive correlations between nesfatin-1 and BMI, percent of body fat and the triceps skinfold thickness [44]. Percent of body fat was demonstrated as the main determinant of nesfatin-1 variance [44]. It seems that our findings consider to significant association between PPARγ level and circulating nesfatin-1 might be consistence with mentioned evidences due to main correlation between circulating PPARγ and fat mass. Another confirming data to justify the markedly correlation between PPARγ and nesfatin-1 demonstrated by Yamada et al. research that interestingly, nesfatin-1, derived from the precursor peptide, NUCB2, from a troglitazone, PPARγ ligand, induced cDNA library [46].

Progranulin has been introduced as an adipokine inducing insulin resistance and obesity [9]. Although, current study's findings demonstrated the main association between this adipokine and circulating PPARγ, but to our knowledge, there is not supporting information to explain observed correlation.

According to some evidences, there are possible mechanisms that influencing diseases related to obesity and therapeutic opportunities for them through PPARγ and several adipokines such as vaspin and RBP4 [16]. There is evidences that confirmed an association of increased circulating RBP4 levels and the metabolic syndrome [47]. There are evidences that RBP4 gene expression is induced in brown fat from mice treated with PPAR agonists. PPARγ can also induce the RBP4 gene in white adipocytes. As a result, it has been concluded that PPARγ-mediated signaling controls RBP4 gene expression through dependent mechanism to PPAR and its co-activators [48]. In view of that, the observed marginal correlation between PPARγ and RBP4 might explain via referred mechanisms from Rosell et al. [48] study.

Plasma omentin-1 levels, were correlated inversely with obesity [49]. However, results of clinical study demonstrated the pioglitazone as PPARγ agonist reduced omentin-1 levels in women [50], but we couldn’t find the significant correlation between circulating PPARγ and omentin-1 levels.

Vaspin, visceral adipose tissue-derived serpin, was originally identified as an adipokine, which were found to be associated with obesity in humans [51]. Evidences from previous studies demonstrated that vaspin mRNA increased with treatment of PPARγ agonist, pioglitazone [2]. We found the marginal positive correlation between PPARγ and this adipokine. Similar to PPARγ, it has been suggested that vaspin might be the compensatory molecule in the pathogenesis of MetS and vaspin-mimicking agents or vaspin recombinant protein might have beneficial effect on MetS improvement [2]. So, it appears all of studied adipokines might have association with PPARγ level and might simultaneously be involve in some common pathway to make susceptible obese subjects for MetS and other obesity related condition. In this regard, there are compelling evidences from previous studies for supporting some of our findings, and need to more research to describe new findings in current study.

A number of caveats need to be considered in interpretation of present findings. Briefly, the relatively small sample size might limit the power to detect precise association between some adipokines and PPARγ. In addition, confounding might exist and influence our analyses. However, we did adjust for the important factors that may affect circulating PPARγ. We acknowledge experimental studies that replication in a diverse population is required to verify our findings.

## Abbreviations

MetS: Metabolic syndrome; PPAR: Peroxisome proliferator-activated receptor; PPARγ: Peroxisome proliferator-activated receptor gamma; TZ: Troglitazone; DXA: Dual energy X-ray absorptiometry; BIA: Bioelectrical impedance analysis; FFM: Free fat mass; TBW: Total body water; hsCRP: Hyper sensitivity C-reactive protein; ELISA: Enzyme-linked immunosorbent assay; ANOVA: Analysis of variance; SEM: Standard error mean; FBS: Fasting blood sugar; TG: Triglyceride.

## Competing interests

No potential conflicts of interests relevant to this article were reported.

## Authors’ contributions

KM, AH and MD conceived of the study, participated in its design, KM, MRE and AH performed the statistical analysis, KM, FK, SH and AAS-Y drafted and edited the manuscript. KM, SAK and AH conducted the literature search, participated in its design and coordination, AH and MD provided critical input during manuscript preparations. KM and AH performed data interpretation and wrote the manuscript. All authors read and approved the final manuscript.
